# A model for homeopathic remedy effects: low dose nanoparticles, allostatic cross-adaptation, and time-dependent sensitization in a complex adaptive system

**DOI:** 10.1186/1472-6882-12-191

**Published:** 2012-10-22

**Authors:** Iris R Bell, Mary Koithan

**Affiliations:** 1 Department of Family and Community Medicine, University of Arizona College of Medicine, 1450 N Cherry, MS 245052, Tucson, AZ, 85719, USA; 2 University of Arizona College of Nursing, Tucson, AZ, USA

**Keywords:** Homeopathy, Nanoparticles, Silica, Epitaxy, Hormesis, Cross adaptation, Time dependent sensitization, Metaplasticity, Allostasis, Complex adaptive system, Stress response network, Resilience, Nanomedicine

## Abstract

**Background:**

This paper proposes a novel model for homeopathic remedy action on living systems. Research indicates that homeopathic remedies (a) contain measurable source and silica nanoparticles heterogeneously dispersed in colloidal solution; (b) act by modulating biological function of the allostatic stress response network (c) evoke biphasic actions on living systems via organism-dependent adaptive and endogenously amplified effects; (d) improve systemic resilience.

**Discussion:**

The proposed active components of homeopathic remedies are nanoparticles of source substance in water-based colloidal solution, not bulk-form drugs. Nanoparticles have unique biological and physico-chemical properties, including increased catalytic reactivity, protein and DNA adsorption, bioavailability, dose-sparing, electromagnetic, and quantum effects different from bulk-form materials. Trituration and/or liquid succussions during classical remedy preparation create “top-down” nanostructures. Plants can biosynthesize remedy-templated silica nanostructures. Nanoparticles stimulate hormesis, a beneficial low-dose adaptive response. Homeopathic remedies prescribed in low doses spaced intermittently over time act as biological signals that stimulate the organism’s allostatic biological stress response network, evoking nonlinear modulatory, self-organizing change. Potential mechanisms include time-dependent sensitization (TDS), a type of adaptive plasticity/metaplasticity involving progressive amplification of host responses, which reverse direction and oscillate at physiological limits. To mobilize hormesis and TDS, the remedy must be appraised as *a salient, but low level, novel threat, stressor, or homeostatic disruption for the whole organism*. Silica nanoparticles adsorb remedy source and amplify effects. Properly-timed remedy dosing elicits disease-primed compensatory reversal in direction of maladaptive dynamics of the allostatic network, thus promoting resilience and recovery from disease.

**Summary:**

Homeopathic remedies are proposed as source nanoparticles that mobilize hormesis and time-dependent sensitization via non-pharmacological effects on specific biological adaptive and amplification mechanisms. The nanoparticle nature of remedies would distinguish them from conventional bulk drugs in structure, morphology, and functional properties. Outcomes would depend upon the ability of the organism to respond to the remedy as a novel stressor or heterotypic biological threat, initiating reversals of cumulative, cross-adapted biological maladaptations underlying disease in the allostatic stress response network. Systemic resilience would improve. This model provides a foundation for theory-driven research on the role of nanomaterials in living systems, mechanisms of homeopathic remedy actions and translational uses in nanomedicine.

## Background

**Figure 1:**
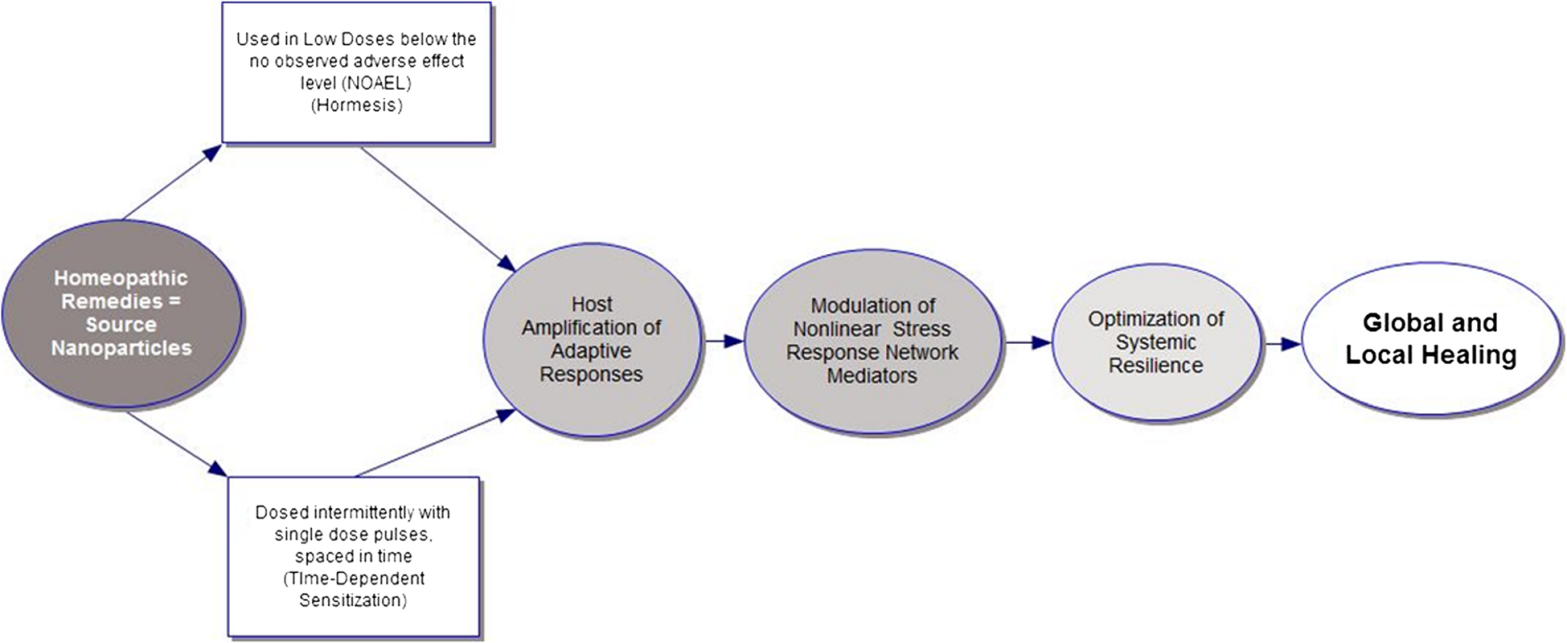
**Nano particle model for homeopathic remedy action: hormesis, allostatic cross-adaptation, and time-dependent sensitization of the nonlinear stress response mediator network.** Global and local healing occur across the person as a self-organized complex adaptive system in response to the individualized remedy serving as personalized hormetic stressor, i.e., holistic nanomedicine: an exogenous nanoparticle stimulating self-amplified, bidirectional adaptive change (see text).

The purpose of this paper is to propose a model that explains how homeopathic remedies act on living systems (Figure 1). Basic science research suggests that classically-prepared homeopathic remedies (A) contain measurable source nanoparticles (NPs) and/or silica nanoparticles with adsorbed source materials [1-4] which are heterogeneously dispersed in colloidal solution; (B) act by modulating biological function of the allostatic stress response network [5,6], including cytokines, oxidative stress and heat shock proteins [7,8], as well as immune, endocrine, metabolic, autonomic and central nervous system functions [9,10]; (C) evoke biphasic actions on the adaptive plasticity of living systems [11-15] via organism-dependent, endogenously amplified, rather than agent-dependent pharmacological, effects [16]. The effects of homeopathic remedy nanoparticles involve state- and time-dependent adaptive changes [7,8,17-20] within the complex adaptive organism [19-22]. The main clinical outcome is (D) improvement in systemic resilience to future environmental stressors and recovery back to normal healthy homeostatic functioning [23]. Disease resolves as an indirect result of changing the system dynamics that had supported its original emergence [21,22], rather than as a direct result of suppressing end organ symptoms.

Other investigators have proposed a variety of theories for homeopathic remedy effects, e.g., persistent memory of unique water structures, water-ethanol clusters, epitaxy, and nanobubbles [24-32], glass-derived silica crystals and structures [4], electromagnetic activities [33], biological signaling [9], quantum macro entanglement [34,35], nonlinear dynamics of complex systems [13,19,20], stressor effects and hormesis [36-38]. The current nanoparticle-cross adaptation-sensitization model incorporates and builds upon many conceptual points and empirical findings from this previous body of work, while offering an integrated, comprehensive synthesis for systematic testing [39].

Homeopathy is an over 200-year-old system of complementary and alternative medicine (CAM) developed by the German physician Samuel Hahnemann, MD. The field has a well-articulated practice theory [40], extensive case report-based clinical literature [41], high levels of patient satisfaction [42,43], and a growing modern research base [44,45]. Nonetheless, homeopathy has engendered some of the most intense skepticism within CAM, largely over the nature of its medicines (“remedies”). The classical process of manufacturing homeopathic medicines involves trituration in lactose and/or serial dilution in ethanol-water solutions and succussion (vigorous repeated cycles of shaking via hand or standardized mechanical arm pounding on a hard surface) in glass vials containing ethanol-water solutions [40]. Common dilution factors are 1 part source to 9 parts diluent (1/10, decimal, D or X potencies) and 1 part source to 99 parts diluent (1/100, centesimal or C potencies). Original bulk-form source materials are typically plant, mineral, or animal in nature.

Once dilution and trituration steps in lactose and/or succussions in liquid solvents begin, any low potency homeopathic remedy prepared above mother tinctures, i.e., 1X to 23X or 1C to 11C, should theoretically still contain bulk-form molecules of source material as well as source nanoparticles [3,46]. In theory, repeated dilution steps leave progressively fewer and fewer molecules of bulk-form source material in a true solution, until eventually none should persist in solution diluted past Avogadro’s number (6 x 10^23^), i.e., potencies higher than 24X or 12C. Ordinary clinical chemical assays can at best find relatively low numbers of bulk-form source molecules, for remedies at low potencies and none at higher potencies. As a result, conventional medical scientists and chemists reject the plausibility of homeopathy because of the presumptive lack of sufficient bulk-form source material to exert a “usual” pharmacological dose–response effect. In typical clinical pharmacology, lower bulk-form “doses” should exert lesser effects, until there are no biological effects at all.

These points are seemingly valid, if the underlying assumptions are valid – i.e., that homeopathic medicines are ordinary, dissolved and diluted bulk-form chemical drugs in true solution that could only act pharmacologically [47] with linear dose–response relationships. However, the trituration and succussion procedures in classical homeopathic remedy preparation may actually be crude manual methods that generate “top down” nanoparticles of source material. Nanoparticles range in size from 1 nanometer (nm) on a side up to 1000 nm or more, though much nanoscience research focuses on special acquired properties of small nanoparticles below 100 nm [48]. Trituration with mortar and pestle is a manual method for mechanical grinding or milling, similar to ball milling used in modern nanotechnology [49,50]. Like modern nanotechnology methods of microfluidization [51,52], sonication [53,54], and vortexing [55], manual succussions introduce intense turbulence, particle collisions, and shear forces into solution that break off smaller and smaller particles of remedy source material as well as silica from the walls of the glass containers or vials [1]. The combined impact of these mechanical nanosizing procedures [54] would be to modify the properties of the remedy [26,30,32], generating remedy source nanoparticles [2,3], as well as silica crystals and amorphous nanoparticles [3,4,32].

Persistent remedy source nanoparticles have been demonstrated with high resolution types of electron microscopy in metal and plant homeopathic remedies prepared both below and above Avogadro’s number [2,3]. Studies also report finding measurable amounts of nanosilica and its precursors in glassware-prepared remedies and other medicines [3,4,32,56]. The types of glassware [56] (or polymer containers [1]), pH, temperature, amounts of agitation, and the ratio of ethanol to water solvent [57,58] can further modify the specific sizes and properties of the resultant nanoparticles. Nanoparticles are different from bulk-form materials as a function of their small size, including acquired adsorptive [56,59], electromagnetic, optical, thermal, and quantum properties [33,48,60,61].

With their highly reactive and catalytic surfaces [48], NPs aggregate through self-assembly, and readily adsorb other nanoparticles and organic materials onto their surfaces, e.g., DNA, proteins, plant extracts or lactose [48,60,62-68]. In addition to mechanical attrition methods, multiple studies have demonstrated that plant mother tinctures can biosynthesize metal (silver or gold) or silica (silicon dioxide) nanoparticles and structures via natural phytochemical reactions *in vitro*[64,69-72]. When plant herbal tinctures are used for biochemical synthesis of silver or gold metal nanoparticles from metal salt solutions, evidence indicates that the herb adsorbs onto the surfaces and modifies the sizes and properties of the resultant metal nanoparticles during this “green” manufacturing process [64,69,73]. The metal NPs then can convey plant-modified specific biological effects [64]. Such nanoparticles could augment and amplify the more direct, bulk herb-like properties for very low potency remedies made from ethanolic plant mother tinctures [46].

At higher liquid potencies, silica from the glass container walls released during succussions appears to be an important contributor to the generation of active homeopathic remedies [1,3,4,32,74]. Experimental data also show that nanosilica can self-assemble into 3-dimensional structures that can withstand drying, using DNA, proteins, or living cells as biological templates (a type of epitaxy) [71,72,75-78]. The interaction and adsorption of specific remedy source with lactose and/or silica in the lowest homeopathic potencies such as 1C or 1X and the next few very low potencies in glass vials containing ethanol-water solutions would create remedy-specific lactose- [79] and/or silica-adsorbed “nanoseeds” for generating subsequent potencies [3,64].

Once formed at lower potencies, remedy NPs and remedy source-modified nanosilica [64,67,78] could be capable of seeding regrowth or self-assembly of pre-formed silica nanostructures at higher potencies [4,32,71,80]. Nanotechnologists regularly use silica in bottom up self assembly of specific nanostructures based on DNA, proteins, or other materials as epitaxial structural templates [67,77]. Any involvement of silica [3] or other nanostructures [32] would occur in addition to the demonstrated physical transfer of detectable remedy source nanoparticles themselves during the serial dilutions into higher potencies [2]. Nanosilica would serve as a non-specific biological amplifier [81,82], if present [1], as well as a vehicle for additional remedy-specific structural and/or electromagnetic information.

For instance, one type of amorphous nanosilica can retain memory of an electric- or magnetic-field induced orientation [83]. Previous studies have shown that some homeopathically-prepared materials can emit detectable electromagnetic signals [33]. Such signals could, along with the adsorbed and perhaps encapsulated remedy nanomaterial structures [65] and epitaxial processes [3,24,64,71], thereby convey remedy-specific information in these multiple ways. The information could derive from lasting alterations in the electrical conductivity of nanosilica and other nanostructures. The process might take advantage of silicon’s semiconductor capabilities when “doped” with very small quantities of some inorganic or organic materials, i.e., from remedy source NPs in liquid potencies. However, since homeopathic remedies are often dried onto lactose pellets for storage and convenient transport, any model for homeopathy must also accommodate the need to retain the remedy-specific signal while dried and restore it upon clinical administration. Silica and protein nanostructures can survive drying [66,77]. Lactose can absorb intact nanoparticles sprayed onto its surfaces [66].

Detection and study of these particles and proposed nanostructures present scientific challenges. Ordinary chemical assays and light microscopy cannot detect the nanoparticles, especially at higher potencies [84,85]. Certain types of spectroscopy, e.g., Raman [24], but not always others, e.g., NMR [32,86], can indirectly detect their presence in solution. Various physico-chemical methods can find indirect evidence from heat or light release by disrupting the dynamic structures that nanoparticles form in the solvent. High resolution imaging techniques [84,87,88], including atomic force microscopy, scanning electron microscopy, or transmission electron microscopy, can directly provide images of the actual presence of identifiable source nanoparticles in a given remedy [2,3,69]. Technological advances for characterizing single nanoparticles may also facilitate this type of research [88,89]. With their increased bioavailability and reactivity, nanoparticles lower the doses of a drug, herb, nutriceutical, or antigen needed to produce clinical effects in medical applications, by orders of magnitude [63,68,90,91].

## Discussion

### Overview of the model

Three assumptions frame the discussion, and four principles provide the theoretical basis for this model.

The assumptions from the mainstream physiological literature are:

(1) Human beings, animals, and plants are complex adaptive systems or interconnected self-organizing networks [23,92-94];

(2) The allostatic stress response network of nervous, endocrine, immune, and metabolic pathways within the larger network of the organism is a hub that interacts with and adapts to environmental stressors [5,95,96]. Such stressors are any type of exogenous (or endogenous) stimulus that can disrupt homeostatic balance in the human being as an organism [5,94].

(3) Progressive allostatic overload of the adaptive capacity of the organism by higher intensity stressors leads over time to changes in functional set points and dynamic attractor patterns [10] that underlie the emergence of chronic disease. Disease manifests as unique complex, nonlinear, dynamical patterns of maladaptive function, determined by genetic, epigenetic, and lifestyle factors [5,95].

There are four principles of the nanoparticle-allostatic cross-adaptation-sensitization (NPCAS) model that explain homeopathic remedy action:

(A) Homeopathic remedies are highly reactive source and/or remedy-modified silica (or polymer) nanoparticles, not bulk-form drugs [2,3];

(B) Remedy nanoparticles stimulate a complex adaptive response in the organism that begins in the allostatic stress response network, with cascading indirect consequences over time across the entire self-organizing organism. The homeopathic simillimum (clinically optimal) remedy nanoparticles [16] serve as *low level, but highly salient* novel stressors, i.e., specific biological signals for the overall organism [9];

(C) The adaptive plasticity processes that underlie the direction and magnitude of remedy effects on living systems involve nonlinear physiological phenomena such as hormesis, cross-adaptation, time-dependent sensitization and cross-sensitization/oscillation. As a low intensity stressor, remedy nanoparticles stimulate changes in the opposite direction to those of the higher intensity stressors that fostered the original development of disease [16,97,98]. The disease-related maladaptations prime the system [10,39]. Then the correct remedy in low dose elicits reversal of direction of the maladapted responses.

(D) The adaptive changes that the remedy evokes ultimately strengthen systemic resilience. The successfully treated individual can resist and rebound from subsequent challenges from higher intensity homeostatic disruptors of the organism as a complex network, at global and local levels of organizational scale [22].

In the context of medicine [99-101] and complementary and alternative medicine (CAM) [13,19-22], researchers have previously detailed the evidence that living organisms are complex adaptive systems (CAS) or networks of interconnected, interregulated components. Other investigators have extensively addressed the role of the allostatic stress response network within the organism in adaptation, maladaptation, and the development of disease [5,94,95]. This paper will build upon concepts and findings from the CAS and allostasis-adaptation literatures integrating the research on homeopathic remedies and nanoparticle properties with physiological findings on the processes of adaptation and response amplification. The current model will facilitate development of specific, testable hypotheses for theory-driven homeopathic remedy research [39].

### Literature informing model development

Principle (A). Homeopathic remedies are highly reactive source and/or remedy-modified silica (or polymer) nanoparticles, not bulk-form drugs [2,3]; The active components of homeopathic remedies, other than plant mother tinctures, are nanoparticles of the source substance [2] and/or source substance adsorbed to the surface of or entrapped within silica or polymer vehicle nanoparticles [1,3,62-65] in ethanol-water colloidal solution. At higher potencies, bottom-up nanosilica self-assembly and epitaxial templates from remedy source nano-forms encountered during earlier preparation at lower potencies could also acquire, retain, and convey remedy-specific information [3,4,78]. Other than in plant mother tincture concentrates [46], remedies are not purely bulk-form material drugs. Trituration of insoluble bulk form materials, which is mechanical grinding in lactose, would generate source material and lactose amorphous nanoparticles and nanocrystals [49,79]. With or without source bulk-form material trituration, repeated succussions in ethanol-water solutions would generate not only remedy source nanoparticles [2,3], but also silica (or synthetic polymer) nanoparticles and nanostructures from the walls of the glass (or synthetic polymer) containers in which the succussion occurs [3,4,56,64].

Nanotechnology research suggests that variations in a number of different manufacturing parameters, e.g., glassware, solvent, pH, temperature, type of container, grinding methods, force and number of cycles of agitation of fluids, will affect the sizes, shapes, and properties of resultant nanoparticles [57,58,102]. For instance, smaller nanoparticles, e.g., 16 nm nanosilica, are generally more toxic to healthy cells than larger nanoparticles of the “same” material [103-106]. However, compared with smaller NP sizes (e.g., 20 nm), larger size nanoparticles (e.g., 80 nm) of the “same” calcium phosphate source material induce apoptosis more effectively in osteosarcoma cancer cells [107]. Notably, the homeopathic remedy *Calcarea Phosphoricum* in low potencies has long been part of the Banerji treatment protocols for osteosarcoma and other cancers in India [108].

A recent empirical breakthrough in understanding the basic nature of homeopathic remedies demonstrated that even commercial metal remedies (source materials: gold, copper, tin, zinc, silver, and platinum) triturated, diluted and hand-succussed to 30C or 200C potencies (both above Avogadro’s number) retain nanoparticles of their source material [2]. Some have criticized the Chikramane et al. paper for using sample preparation methods that failed to detect size differences of nanoparticles at different potencies [109]. Nonetheless, the overlap of classical homeopathic manufacturing and mechanical top-down nanoparticle nanotechnology manufacturing methods and findings from other basic science laboratories converge with similar findings [3,24,64].

For instance, Upadhyay and Nayak [3] used electron microscopy to demonstrate nanoparticles and nanocrystals in three different homeopathically-prepared plant remedies at 1C through 15C potencies. The latter researchers also measured greater amounts of silicon in succussed than in unsuccussed remedies and water controls made in glass vials. Glass-vial succussed remedies exhibited greater silicon contents than plastic-vial succussed remedies [3], a finding consistent with previous studies [1,4,32,74].

Das et al. [64] recently reported using four different homeopathic plant mother tinctures to biosynthesize silver nanoparticles, whose sizes and associated biological effects differed *as a function of the specific plant used in their manufacturing*. The data imply that interaction of metal nanoparticles with a bioactive material in solution can acquire unique properties as a result. Plant extracts can also induce formation of colloidal silica structures (cf. [71]). Such nanostructures could undergo the same types of remedy source adsorption and size modifications from biochemical interactions with specific plant or animal source materials, as now documented for biosynthesized silver NPs.

In an earlier paper, Rao et al. [24] suggested that the commercial homeopathic remedies they studied contained nanobubbles of oxygen, nitrogen, carbon dioxide, and *possibly remedy source material*, generated from the succussion component of homeopathic remedy preparation. They also proposed epitaxy (transfer of structural but not molecular information) as another mechanism by which specific remedy materials could transfer information to water structures in the ethanol-water solution [24]. Thus, both adsorbed remedy source nanoparticles and specific epitaxial information transferral to silica nanostructures [3] are viable hypotheses consistent with the large literature demonstrating individualized biological and physical chemistry specificity of different homeopathic remedies.

These data contribute to better understanding of reports from two other laboratories studying homeopathic remedies. For example, Elia et al. [26,28] found that extreme changes in pH, e.g., strong alkaline pH, cause homeopathic remedy verums to release measurably excess heat and exhibit higher electrical conductivity compared with control solvents. They suggested that the heat release was energy from disruption of ordered structures in the remedy solutions that was not present in plain solvents.

Although Elia interpreted their findings in terms of changes in water structure [26,28], their data are also consistent with a nanomaterial model. Since several studies have shown that succussion in glass vials releases measurable amounts of silica or its precursors [3,4,56], alkaline pH fosters formation of silica [110], and silica nanofluids exhibit increased electrical conductivity when temperature rises [111], the presence of nanoparticles could account for the increased heat release and electrical conductivity.

In addition, Elia et al. [26] reported that the amount of heat release and electrical conductivity increased when their remedy solutions were tested using extreme pH changes after storage in small volumes for extended periods of time at room temperature. Such findings are consistent with the strong thermodynamic tendency of smaller nanoparticles of remedy source and/or silica in liquid sols to aggregate spontaneously and/or self-assemble back into larger crystalline structures, e.g., via Ostwald ripening, unless specifically treated to prevent this phenomenon [112-115]. In nanoscience, the material composition may not change, but the structural organization and properties can.

Furthermore, Rey [30] found that, under extreme *in vitro* external treatments with low temperatures followed by x-ray and gradual rewarming procedures, higher potency homeopathic remedies released measurably more light energy than did the control solutions. Rey also noted that two remedies differed from one another in thermo luminescence patterns, but retained the “fingerprint” properties of the original source substance, even without the detectable presence of the bulk-form source material in solution. Although Rey discussed his data in terms of the “memory of water” models, the latter findings are consistent with the persistent presence of identifiable remedy source nanoparticles in the verum test solutions [2] and/or residual specific remedy source material and information adsorbed and templated onto silica nanoparticles and silica nanocrystals [1,3,32,64,69].

On the one hand, silica NPs alone could not explain Rey’s ability to use thermo luminescence for distinguishing different homeopathic source materials from one another at lower or higher potencies. Other laboratories have also been able to distinguish one specific higher potency remedy from one another and from succussed solvent controls using Raman and UV–vis spectroscopic methods [24,116], as well as less well-known technologies [117,118]. In contrast, NMR spectroscopy and infrared spectroscopy on different remedies have yielded both positive [32,119] and negative results [86] distinguishing specific remedy solutions from controls. On the other hand, persistent remedy source nanoparticles and remedy-modified silica nanostructures could provide an alternative explanation of Rey’s findings of source-specific information. It is a step forward to recognize that the remedy source information would be present starting in very low potencies not only as bulk-form material, but also as source nanomaterial, while serial dilutions and succussions proceed toward higher potencies. Even if the bulk-form materials might be progressively diluted out of higher potencies, the evidence indicates that the remedy nano-forms and/or their information persist [2,3,9].

Recent homeopathic research contributes to insights about minimally necessary factors to make biologically active remedies. For example, trituration of *Arsenicum Album*, followed by dilution without succussion up to a modified 200C, could still generate biologically active medicine [120]. However, dilution without prior trituration or subsequent succussions of cytokines, produced much less biological activity than succussed forms of the “same” agent [121]. These findings suggest that either trituration or succussion is minimally essential for an active homeopathic remedy; each procedure would mechanically generate nanoparticles [51].

Optimally, however, as Hahnemann reported in combining trituration and succussion to prepare homeopathic remedies [40], nanotechnologists have also found that combining mechanical wet grinding with sonication (agitation in liquid solvent) is more effective than either method alone for forming, de-aggregating, and dispersing nanomaterials [54]. Increased dynamic solute aggregation can occur in aqueous solutions prepared with more versus less dilution, that is, lower initial solute concentrations [122]. Thus, the cumulative shear forces and greater de-aggregation from the additional succussions done while making increasingly higher potencies might translate into smaller sized remedy nanostructures.

Variability in nanoparticle sizes, shapes, and associated properties [103,104] would contribute to the known variability in clinical responses to a specific homeopathic remedy dose. It also explains some of the reproducibility challenges that have been identified in the literature [123,124]. These issues do not negate the validity of homeopathy; rather, they suggest theory-driven directions for systematic research on the variability in remedy nanoparticles, the potential NP contributions to variability in experimental reproducibility in homeopathy, and new ways to evaluate and control specific variables involved in manufacturing methods [1,2] and safety assessments [45,125].

Given the growing body of empirical evidence about the nanoparticle nature and biological activities of homeopathic remedies [2-4,7-10,123], it is time to question the conventional assumption that homeopathic remedies are “simply” dilutions of ordinary bulk-form drugs containing “nothing” but unmodified alcohol and water. The actual evidence suggests that homeopathic remedies are low doses of different sizes and shapes of nanoparticles and nanocrystals of their specific remedy source as well as silica nanostructures with remedy source material adsorbed to their surfaces [2,3]. In this context, even higher remedy potencies retain source-specific structural and electromagnetic “memory” of information within (i) the nanoremedy NPs and (ii) nanosilica structures [64,83] that initially would self-assemble in “bottom-up” aggregates [67,76,77,126,127], around the remedy source materials as structural (epitaxial) templates in solution [122]. Thus, as an alternative to the “memory of water” debate surrounding homeopathy, an empirically-grounded hypothesis would be the “memory of source and source-modified silica nanostructures.”

The organism treats many types of exogenous nanoparticles, including nanosilica [81,82,128,129], as threats to its survival. Local cellular interactions with NPs can lead to systemic signaling [130]. NPs from a salient homeopathic remedy in potency could act mainly as a *novel, low level threat or exogenous stressor,* signaling danger that sets adaptive responses into motion within the physiological and biochemical dynamics of the whole organism [94,128]. Homeopathic remedies would thus act more as low level triggers for systemic stress responses than as pharmacological drugs on specific local tissue receptors [8,37].

Principle (B). Homeopathic remedy nanoparticles, administered intermittently over time [40], act as biological stressors that signal [9] a low intensity novel “threat” to the allostatic stress response network.

### * Allostatic stress response network*

**Figure 2:**
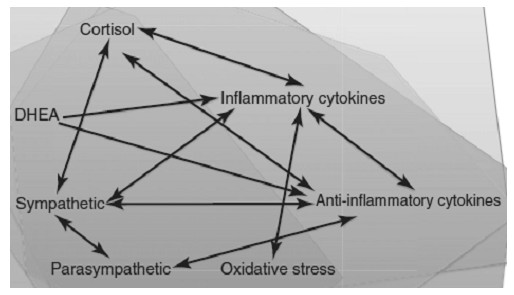
**Nonlinear allostatic stress response network: interface with environmental stressors, including homeopathic remedy nanoparticles.** This schematic shows some of the physiological components involved in the organis’s response to stress and the complex, nonlinear interrelationships as a network within which they regulate one another. Pathways in the central nervous system, including amygdala, prefrontal cortex, and hippocampus, involved in stress responsivity and reward, learning and memory, somatosensory function, emotional function, and motor activity regulate and interact with all of the above components. Disease is an emergent outcome when the cumulative stress load overwhelms the adaptive capacity of the system and the interactions become persistently dysregulated. Targeted, timed disruption of the dysfunctional dynamics of disease affords the system an opportunity to recover normal regulatory relationships and interactions across the biological network [23]. The present model postulates that the correct homeopathic remedy provides such a disruption to initiate adaptive changes. Used with permission from [5].

The immediate interface between the environment and the organism is the allostatic nonlinear stress response network (Figure 2). In the context of physiology, a stressor is any type of environmental or exogenous stimulus or signal that perturbs the system’s homeostasis and sets compensatory adaptive changes into motion. The range of stressors can include biological, infectious, chemical, physical, nutritional, electromagnetic, and/or psychosocial types, i.e., changes that constitute a perceived threat to the survival of the organism. A lower intensity stressor that stimulates adaptive plasticity and regulates the system bidirectionally to restore homeostasis is acting on endogenous processes of adaptation. Well-chosen homeopathic remedy nanoparticles (simillimum) act as deep-acting plasticity-modifying signals in chronic diseases. In nanoparticle form, remedies effect these changes by modulating genetic pathways as well as the nonlinear dynamical function of biological defenses in the organism as a complex system [9,131-133], shifting from a maladapted attractor pattern to a healthier attractor pattern [10,20,21].

In a complex adaptive system, the allostatic network initiates plastic and metaplastic adaptations that evolve over time, preparing the organism to maintain and/or restore homeostasis more effectively in the future if and when it next encounters a similar stressor/signal. These biological signals must be spaced over time to avoid overwhelming the organism’s compensatory capacity. McEwen [134] emphasized the primary role of the brain as one of the controlling hubs in the human being as a complex organism, intercommunicating with bodily cells and regulating the rest of the stress response network.

In an intact complex organism, cells would send signals to the brain via the allostatic network [135,136], e.g., cytokine activation patterns [137]. Perception and processing of environmental threats from exogenous stressors occurs in prefrontal cortex, hippocampus, and amygdala [6,138]. In this context, the salient remedy signals those brain regions with a perceived low level threat to survival of the organism. Consistent with this conceptualization, a previous homeopathic research study demonstrated unique prefrontal EEG cordance reactions to sniffing individualized remedies in human subjects [139]. However, remedies can also initiate responses at lower local levels of organizational scale. Thus, isolated cells as biological systems can detect and respond to environmental stressors [93,140-142]. However, *in vivo*, cells and organism maintain a bidirectional, interactive influence on one another [143]. Local cellular changes send biological signals to the larger system in which they are embedded, and vice versa [94,130].

Changes in the function of such a major network within an organism by necessity induce changes in other physiological networks with which it interacts. In turn, changes in bodily networks interact bidirectionally with the emergent global properties of the organism as a whole [101,143]. Thus, when remedy nanoparticles signal a novel environmental threat to the immune system [10], brain and/or other components of the stress response network, their effects are indirect and magnified over time *by the organism*[21,23,94].

The nonlinear allostatic stress response network includes not only the immune system and brain, but also other interactive, mutually regulatory mediators of adaptation: e.g., cortisol, parasympathetic and sympathetic branches of the autonomic nervous system, metabolic hormones, and biological mediators such as inflammatory and anti-inflammatory cytokines [5]. In the allostatic model of disease, the cumulative overwhelming effects of previous stressors (biological, infectious, physical, electromagnetic, chemical, nutritional and/or psychosocial) initiate persistent dysregulations in the biology of the organism’s stress response network. This allostatic overload causes adverse changes in functional set points, from which the body is unable to recover on its own. The process creates chronic physiological imbalances and permissive conditions for cumulative damage that manifest with symptoms of disease [5,95,144]. These chronic changes in functional set points would correspond in the terminology of complex adaptive systems to becoming “stuck” in a more rigid and less adaptive dynamical attractor pattern [10,20,23,145].

This aspect of the model has support in the basic science research literature on homeopathic remedies. Remedies can mobilize various elements of the allostatic stress response network *in vitro* and *in vivo*. Previous empirical studies have shown that different homeopathic remedies modulate components of the allostatic stress response network. These findings include remedy-induced changes in heat shock proteins [7,17,146], cytokines [147-149], immune [150-153], metabolic [12,131], and nervous system [154-162] function, as well as gene expression patterns [9,163,164]. Nanoparticles per se can and do mobilize components of the allostatic network [165-167]. Because of the interconnected network nature of the allostatic network, however, *in vivo* studies, which allow the brain and body to carry on their usual bidirectional homeostatic interactions with one another, are most likely to capture the hypothesized role of the allostatic network in adaptation [96] after remedy administration.

The specific pattern of the biological responses depends in part upon which components of the stress response network are initially involved [168,169]. For instance, an infectious agent or environmental nanoparticles would likely interact first with elements of the immune system [165,167], but then the cytokines released as part of the immune and inflammatory response in the allostatic network would modulate brain function, leading to changes in emotional state, mood and energy levels [136,170]. In the “other” direction from above downward in the stress response network, chronic disturbances in brain function such as sleep deprivation can mobilize sympathetic nervous system tone, inflammatory cytokine release, and glucocorticoid activity [171]. If any function begins to go up or down, other components of the network will mobilize to regulate and modulate the extent of the change.

###  *Possible mechanisms from stress signaling capabilities of remedy NPs*

The proposed endogenous process begins with the homeopathic remedy nanoparticles serving as novel stressors that signal a salient, organism-wide threat. Skeptics might argue that even with the nanoparticle finding, the quantities are “too low” to make a difference *as direct conventional pharmacological agents acting at local receptors*. However, “low” doses of nanoparticles, which are inherently highly bioactive and catalytic, can be very low and still elicit meaningful biological responses as biological signals for adaptive changes [7-9,16,131,172,173]. Chikramane et al. [2] found measurable quantities of metal remedy source in the nanoparticles they observed from commercially-prepared, hand-succussed remedies were in the range of 1 to 4000 picograms/ml (approximately 0.05 to 200 pg in a one drop liquid dose). For perspective, one picogram of genetic material from a virus (nanosize 10–150 nm), depending on the virus, could contain approximately 1,000,000 or more virus particle equivalents [174]. Physiological levels of various hormones are in the pg/ml range.

How would remedy nanoparticles convey their source-specific information to the allostatic network of the organism? Once generated, nanoparticles can signal specific information about their entry into the organism via their altered size- and shape-dependent chemical, optical, electromagnetic, magnetic, thermal, and/or quantum properties [33,48,60-62,175]. Data from the nanoparticle research literature suggest multiple non-exclusive options: (i) Remedy nano-forms inherently lower the necessary dose levels [90] because of their enhanced bioavailability, intracellular access [90], and biological signaling effects [130], e.g., for plant and mineral source materials; (ii) Silica NPs and crystals act as cellular stressors [128] and adjuvants, i.e., non-specific biological amplifiers [81,82], to stimulate immunological and/or inflammatory reactivity on their own [130] or to source-specific NPs or antigen [176]. Remedies modulate specific genomic expression patterns as well [9,163,164].

In addition, quantum phenomena emerge in extremely small sized NPs with more atom-like characteristics (e.g., <50 nm) [48]. Chikramane et al. [2] reported homeopathic metal remedy NP sizes in the range of 5–10 nm, with the majority of crystallites below 15 nm in size. Thus, some remedy source NPs and/or silica nanostructures templated onto source materials during initial manufacturing steps might also convey remedy-specific information into higher potencies via quantum macro entanglement effects [61] in living cells [177]. Rather than treat the evidence for quantum phenomena in homeopathic remedy testing as a curious anomaly [34], the present model allows the possibility that the quantum mechanical properties of very small nanoparticles [61,178] could further explain some data on homeopathic remedies and variability of experimental reproducibility with placebo-controlled study designs [34,44].

By whatever mechanism(s), the most immediate locus of action of the correct homeopathic remedy would be the allostatic stress response network. The remedy nanoparticles would serve as a significant and salient exogenous danger signal that stresses and thus perturbs the organism’s physiological and biochemical dynamics (cf. [179]). The state of the organism at the time of dose administration is an essential factor in the magnitude and direction of the effects. If the dynamics are currently dysfunctional or diseased, the low dose remedy-elicited perturbation “unsticks” the system [10,20,145]. The disruption provides the system an opportunity to adapt [93] – i.e., to readjust its processes [23], engage its plasticity [16,23,180-182], reverse direction, and restore healthier balance across its global emergent function and local network components [23,93,181,183]. In the pharmacology and physiology literatures, this beneficial adaptive process is termed hormesis.

Principle (C). The mediating processes for remedy effects are physiological, not pharmacological [184]. They include adaptive plasticity and metaplasticity [181,185] of the organism to amplify [169] and modulate the direction of its responses to a salient homeopathic remedy with the passage of time [23], as a function of the organism’s past history [186,187].

### * Hormetic changes*

Mattson [188] defines hormesis as follows: “Hormesis is a term used by toxicologists to refer to a biphasic dose response to an environmental agent characterized by a low dose stimulation or beneficial effect and a high dose inhibitory or toxic effect. In biology and medicine, hormesis is defined as an adaptive response of cells and organisms to a moderate (usually intermittent) stress.” *Hormetic effects are nonlinear and depend on specific adaptive changes in the organism, not on specific pharmacological effects of the substance*[184]. Nanoparticles can cause hormesis [16].

Of relevance to the low doses used in homeopathic treatment, the small size and heightened reactivity of nanoparticles [60] that increase bioactivity and bioavailability of drugs, antioxidants, and herbs [62,63,68] would downshift the hormetic dose–response range even lower [39]. As a result, the small quantity of remedy needed to produce an effect – in nanoparticle form – would fall into the hormetic range [12], potentially far below the already low doses at which this phenomenon usually occurs for bulk-form materials [189,190].

The evolutionary advantage of low dose stimulation rather than inhibition of function is postulated to be the survival advantage conferred on the organism [191]. The compensatory changes in response to the low dose exposure pre-adapt the organism in a manner that will make it more resistant to a repeat danger from the same stressor, or a cross-adapted stressor [8], at an even higher, more toxic or lethal potential dose level [192]. At the same time, living systems self-regulate within a relatively narrow range of function to maintain homeostasis. For example, if certain brain neurons of an organism have a lowered threshold for firing after responding to a given stimulus, then the next stimulus would raise the threshold, and vice versa [181]. As a result, low intensity stimuli can activate, and high intensity stimuli dampen, responses [181].

###  *Cross-adaptation*

Cross-adaptation is a well-documented physiological and biochemical phenomenon [168,169,182,193,194]. In cross-adaptation, unrelated types of stressors, e.g., hypoxia versus cold temperatures, can affect the same intermediaries in the biological allostatic network [7,8,187,193]. That is, although two types of environmental stressors can be quite different in nature, the organism mobilizes the same set of adaptive changes and subsequently copes better physiologically with both stressors [168,182,193,194]. Living systems have a broad, but nonetheless circumscribed, repertoire of possible behaviors in response to environmental challenges. Evolutionary efficiency may have left the organism with the ability to prepare itself against a range of future stressors by initially adapting to one type of stressor [191].

The direction of changes in cross-adaptation can be bidirectional. That is, a given environmental stressor can cause adaptive changes in the organism that make it more or less fit to resist the adverse effects of a different type of stressor [181,182,195]. As in hormesis, low intensity stressors often produce adaptive changes in the opposite direction to high intensity stressors of the same or different type [16,98]. The direction and nature of the response depends on the initial conditions of the organism, past history of the organism, the pattern of adaptive responses that the specific stressor can evoke, and the capacity for adaptation that the organism can achieve [169]. Cross-adapted responses involve the same compensatory mechanisms in the body that the individual’s cumulative past specific stress load has already primed and modified.

When the salient homeopathic remedy serves as a low-level novel but cross-adapted stressor, salutary effects would evolve over time because of pre-existing adaptations to disease-related stressors that the organism had already developed. In the current model, the remedy nanoparticles would mobilize a biological cross-adaptation response [168,169,182,194] to the net effects of the original stressors that previously led to the disease state. The direction of changes to the remedy in the organism, however, would be opposite to those of the higher intensity stressors that originally caused disease. That is, well-matched homeopathic remedy nanoparticles mobilize cross-adaptation within the same allostatic network components that the individual’s disease had previously affected.

Thus, a homeopathic clinical profile would need to match global and local symptom patterns and modalities of expression for the correct remedy (simillimum). The simillimum for an individual patient who is made worse by the approach of a storm or hot temperatures (e.g., particular physical generalities or modalities in the homeopathic clinical literature -[196]), must be a novel stressor with the ability, at higher doses, to evoke a similar, specific set of physiological adaptations to dropping barometric pressures or hot environmental temperatures [182,194,195]. A remedy whose source material bulk form has no effect either way on the specific adaptations [195] needed to restore homeostasis during the approach of a storm or in hot temperatures would be less likely salient – i.e., therefore less clinically active -- for such an individual.

The nature of the responses that the correct remedy can elicit in the organism is similar to the nature of the dysfunctional responses that the previous stressors initiated as allostatic maladaptations [95]. As the remedy nanoparticles were not the original causative agent for disease, researchers would consider it to be a “heterotypic” hormetic stressor for the organism [8,169]. The prior experience with stressors involved other types of higher intensity stressors that can cross-adapt with the remedy effects on the physiology and biochemistry of the stress response network [187,197]. In short, a well-chosen homeopathic remedy can cause the same adaptational symptom pattern as the disease-causing stressors at high doses, but it acts in discrete low doses as a novel, i.e., heterotypic or heterologous hormetic stressor [10,198].

###  *Metaplasticity and time-dependent sensitization*

Metaplasticity, i.e., the plasticity of plasticity, involves activity-dependent cellular and molecular priming mechanisms that initiate long-lasting changes in the expression of subsequent neuronal plasticity [181]. This priming process occurs in neural networks involved in regulating learning and memory, including addictions [181], as well as emotion [199], somatosensory perception [200], and movement [201]. These pathways include prefrontal cortex, hippocampus and amygdala [199,202]. Changes in neuronal excitatory amino acid receptors such as N-methyl-D-aspartate (NMDA) receptors are key participants in metaplastic mechanisms as well as in neuronal damage after injuries to these brain regions. Glutamate is an exemplar excitatory amino acid that affects NMDA receptors. Notably, Jonas’s research team previously demonstrated that low doses of homeopathically-prepared glutamate can attenuate or reverse direction of adverse effects from high dose glutamate exposure in neuronal cells [160,203].

One stimulus/stressor initiates the metaplasticity; the next stimulus/stressor (the same or a cross-adapted stimulus) elicits plastic responses *modified by the history that the organism had with the original stimulus*. Pushing the system toward its limits leads to a propensity to reverse direction in encounters with subsequent stimuli [186]. One example is the Bienenstock-Cooper-Munro rule for experience-dependent plasticity, in which low level cortical activity increases, whereas high level cortical activity decreases, synaptic strength of active neuronal connections [181,204]. Metaplastic changes can occur at stimulus levels below those needed to elicit observable plastic changes and persist long after exposure to the original stressor ends. Low level and high level stressors can initiate metaplastic changes in opposite directions [98,181].

Time-dependent sensitization (TDS) is a form of metaplastic adaptation that generates progressive endogenous response amplification with the passage of time between repeated, intermittent stimuli or stressors. Homeopathic remedy nanoparticles as stressors for the cells and overall organism would be capable of initiating and/or eliciting TDS. As in any type of neuronal plasticity phenomenon, the initiation and elicitation steps of TDS are activity-dependent. After the initiating exposure, the system prepares itself with compensatory changes that amplify over time -- “a sensitized defense response, enabling it to react faster and/or more strongly, should it ever reencounter the same or a similar stimulus” [187]. The novelty of the pulsed or intermittent quality of the dosing regimen for the organism is essential to set endogenous amplification responses into motion; continuous or ad lib exposures do not mobilize sensitized states [187,205]. In TDS, the initiating and eliciting stressors or drugs must also be individually salient for the organism [97,187,206].

**Figure 3:**
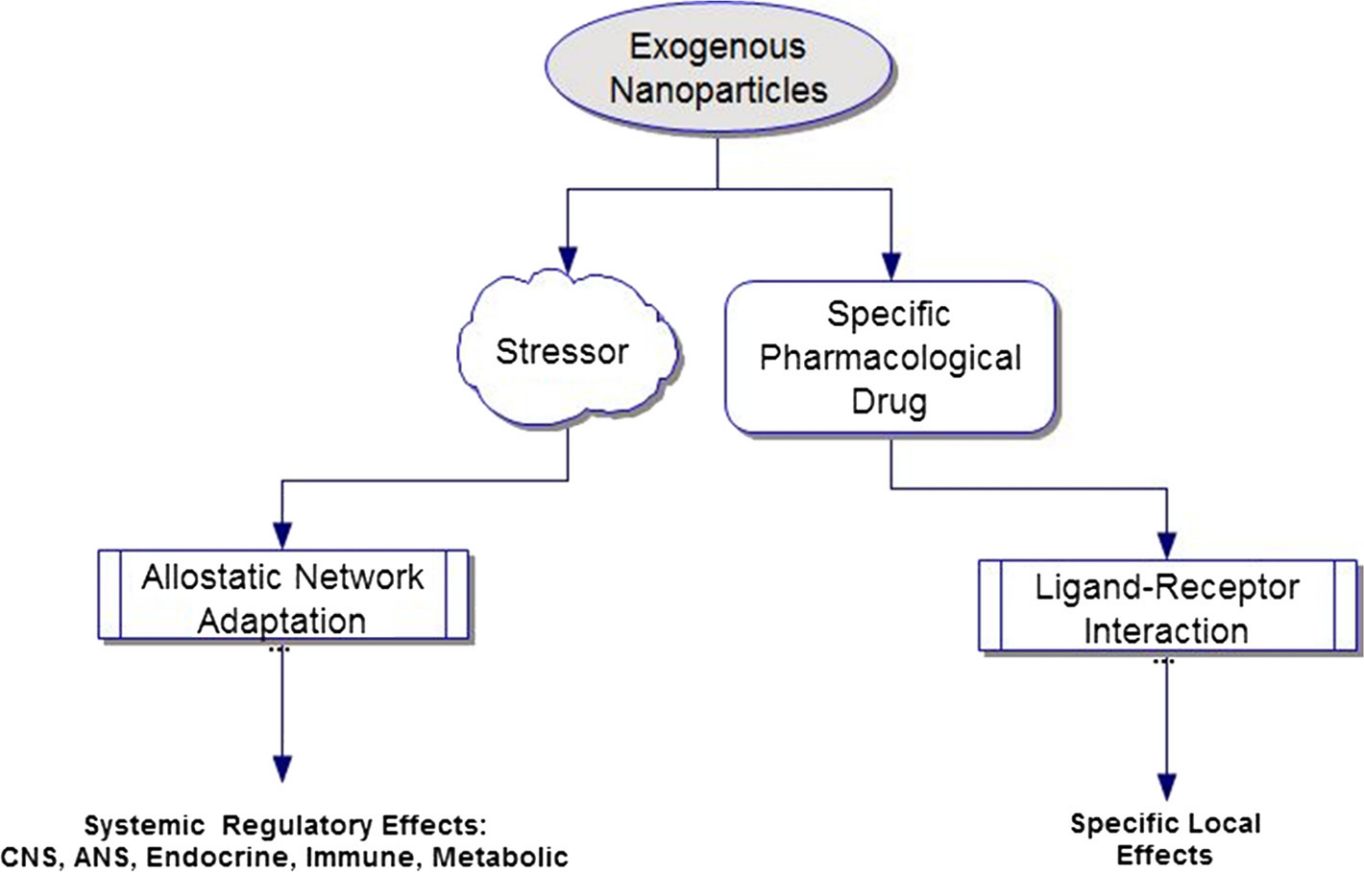
**Dual possible pathways (stressor and/or pharmacological drug) for exogenous agents, including nanoparticles, for effects on living systems.** Exogenous nanoparticles (from pollution, nanomedicine drugs, or homeopathic remedies) might serve as stressors to trigger adaptation and/or specific pharmacological agents (drugs, toxins) to activate specific receptors on local tissues, at higher dose levels. However, although the dose would sometimes be insufficient to act as a specific conventional pharmacological drug, the low levels of nanoparticles found in a homeopathic remedy could act as stressors for the organism (homeostatic disruptors). Therefore, homeopathic remedies would engage mainly the stress response network pathway, whereas conventional bulk form drugs affect both pathways (stressor and drug).

For hormesis [16,38], cross-adaptation, and/or TDS [98,187] to occur in response to a homeopathic remedy dose, the remedy must be perceived or experienced as a salient but low level foreign threat or novel biological stressor, i.e., a potential disruption of homeostasis for the *organism as a whole*. Changes in glucocorticoid hormones and corticoid receptors, major components of the allostatic stress response network, are a necessary but not sufficient early condition for the initiation of TDS [207-209]. For a remedy to be clinically effective, its salience is not to the end organ local mechanisms of symptoms (pharmacological), but rather to the intermediary adaptations in the allostatic stress response network components of the organism (physiological). *The growth in magnitude of the response derives from TDS-based amplifications in the physiological adaptations of the organism, not directly from the size of the initiating stimulus or stressor.* Figure 3 summarizes the role of the organism in experiencing nanoparticles as exogenous stressors and/or pharmacological drugs (or toxicants).

###  *Cross-sensitization*

Like cross-adaptation, cross-sensitization of amplified responses also occurs. Chemically-unrelated agents, e.g., stress and amphetamine or cocaine [209], sucrose and stimulant drugs or alcohol [210,211], formaldehyde and cocaine [212], stress and morphine [213], stress and diazepam [214], can all cross-sensitize with one another. One agent initiates and a different agent elicits sensitized responses. Antelman interpreted these ubiquitous cross-sensitization findings to indicate that the shared feature of the drugs, food, or environmental stimuli was their quality as novel and threatening stressors, i.e., individually salient “danger signals” for the organism, rather than their pharmacologically specific actions [187]. Given the interdisciplinary nature of the present model and discipline-related variations in terminology, the neuroscience concept of cross-sensitization also overlaps that of “heterologous priming” a term from a more immunologically-oriented perspective [10], or “heterologous post conditioning hormesis,” a term from pharmacology-toxicology and physiology [7,189].

Repeated intermittent episodes of exposures to the same or a cross-sensitized stressor can elicit progressively larger responses with the passage of time in TDS [169,187,206]. However, at metaplastically-primed physiological limits, sensitized responses will change direction (oscillate) with each successive dose [97,186,187], thereby potentially promoting recovery from disease *if chosen for salience and properly timed*[11]. As previously noted, in physiology [169,181,215] and behavioral sensitization studies of non-homeopathic stressors and drugs [97,98,216-218], the low versus high dose and the state of the organism interact to produce polar opposites in the direction of the response to the “same” stressor or stimulus.

Together with cross-adaptation, these related phenomena of TDS cross-sensitization and metaplastic oscillation would help explain the clinically-reported ability of a homeopathic remedy to reverse chronic individualized maladaptive patterns in the organism. That is, the remedy NPs are not only cross-adapted, but also cross-sensitized to the overall disease-related dysfunctional changes that are previously established in the organism at the moment of remedy administration. The disease is a sensitized emergent set of previously-amplified dynamical behaviors (allostatic maladaptations) that the body accumulated in response to past higher intensity stressors of all types. These cumulative allostatic disturbances manifest as dysfunctional biological dynamical patterns, induced while the organism was trying unsuccessfully to cope with overwhelming cumulative stress effects of adverse childhood experiences, past life traumas, infections, environmental chemical pollutants, physical stressors, psychosocial stressors, poor nutrition, and/or various other epigenetic factors [5,95,134].

In the present model, a low dose of the correct remedy nanoparticles would push the overloaded allostatic network to its metaplastically-primed physiological limits, which might induce a transient worsening of symptoms, i.e., homeopathic aggravation, before a reversal of direction of the sensitized response occurs [219]. A true aggravation reportedly includes a global sense of increased well-being, suggesting central nervous system involvement at the onset of the remedy response, even though local physical symptoms may temporarily flare, sometimes in association with an acute onset infection [220].

Alternatively, the correct remedy NPs could arrive in the organism at a point when the system dynamics are already disease-primed to a critical dynamic point or maximum physiological limit [8,23]. In the latter case, the reversal in direction from disease toward healing ensues without transient symptom aggravation. Conversely, if the remedy is given to a healthy organism, its metaplastic effects evolve in the direction of enhancing, not reversing, disease-related adaptations [11]. This history- and state-dependent type of variability in response direction and amplitude is well-documented in the physiological literature on adaptation, cross-adaptation, metaplasticity, and cross-sensitization in complex adaptive systems [181,182,186,187,195,216].

Homeopathic clinical research provides support for the involvement of TDS. Convergent research indicates that sensitization of the central nervous system pathways related to pain is a key mechanism in fibromyalgia (FM) [221]. In persons with FM [222], we found that repeated intermittent doses of individualized homeopathic remedies initiate progressively sensitized (amplified) responses of electroencephalographic alpha activity [154], with specific unique changes over time in prefrontal electrode sites for the responders with both global health and local pain improvements [223]. In persons with a mild form of multiple chemical sensitivity, an FM-related condition also tied mechanistically to TDS, repeated intermittent sniffs of an individually-salient homeopathic remedy can induce short-term EEG alpha effects that are nonlinear and even oscillatory in directionality [14].

As types of metaplasticity, both hormesis and TDS mobilize adaptive or compensatory changes in an organism [23] in response to the appraised threat from novel or foreign stressors, biological agents, chemicals, physical stressors, and/or drugs, including nanoparticles [16]. These organism-based, nonlinear adaptive changes evolve separately from any direct specific pharmacological actions on receptors, do not require the continued presence of the initiating agent, and are thus pharmacologically “nonspecific” [7,8,187]. Rather, the organism-based response pattern is specific to the past history and initial state of the organism, the passage of time, and the timing of repeat doses [5,16,23,187,191].

### * Pulsed dosing regimens in modulating cells and organisms as nonlinear dynamical systems*

As in TDS, classical homeopathic practice theory suggests the value of using discrete pulses of a remedy (i.e., “nanoparticle”) dosed at very low quantities in a timed manner as biological signals to initiate healing and stimulate the self-reorganization of the organism [224]. However, current mainstream studies are still focused on using nanomedicines in relatively higher amounts, as if they were conventional drugs to force a direct local action, requiring continuous blood levels, rather than intermittent pulsed dosing. Still, nanoparticles reduce the total amount of a drug or herb needed to produce a given effect [63,225].

The distinction for homeopathy is that the low level nanoparticle exposures occur at discrete points in time, pulsed doses in the salutary low dose hormetic range. The therapeutic intervention involves small quantities of nanoparticles, (a) selected for salience to the individual’s unique emergent maladaptive pattern, and (b) given in discrete pulses at widely spaced intervals of time as organism-specific stressors that evoke an endogenous cascade of adaptive changes [224]. Without the remedy NPs as a discrete low intensity eliciting stimulus, the organism’s metaplastic priming would not express itself.

Research on dynamical diseases in complex adaptive systems has shown that pulsed, properly-timed stimuli can interrupt the dynamics of a pathophysiological process such as a seizure [226] or a cardiac arrhythmia [227] and cause the affected system to revert to normal function [228]. Once an interconnected part of a complex system or network changes its dynamics, the alterations will then cause additional, albeit indirect, changes distant in space and time from the site of the original stimulus [20,23]. Complex networks undergo recurring mutual interaction patterns (motifs) between global and local organization and function [93,94,142,143].

The pulsed intervention strategy allows the bodily networks to respond to the remedy nanoparticle stimulus, and gives the system time to incorporate changes that further inform changes and adaptive responses [21,22]. Similarly, TDS also requires intermittent stimulus dosing for its initiation and evolution [229], thereby allowing the system time to complete the endogenous amplified adaptive changes following each dose [230-232], before giving the next dose. In sensitization, a drug or biological substance acts as an environmental or exogenous stressor to disturb homeostasis and initiate adaptive responses that amplify with the passage of time, without the continued presence of the initiating agent [187,233]. The capacity for nonlinear amplification of effects from an initially small stimulus in a complex living system such as a human being is well-established [23]. Thus, the rationale for the pulsed dosing regimen relates to the role of the correct remedy in stimulating endogenous adaptive changes to an exogenous stressor, not in its use as a pharmacological agent.

Principle (D). Successful homeopathic treatment strengthens systemic resilience.

Resilience in a system permits it to bounce back to normal function on its own after the impact of a given environmental stressor or challenge [23]. The successfully treated individual can resist and rebound from subsequent challenges from higher intensity homeostatic disruptors of the organism as a complex network, at global and local levels of organizational scale [234].

In complex adaptive systems terms, a resilient system is able to function well within the fitness landscape or environment in which it is embedded [235]. Faced with change, a healthy organism is flexible and able to make further adaptive changes to bring itself back to homeostasis and normal functionality in the context of the modified environment [23,236]. Of course, overwhelming change or a hostile environment might again cause allostatic overload, adverse shifts in functional setpoints, and recurrent disease, necessitating further homeopathic treatment.

Because of the interactions and interdependence of subnetworks within a person’s larger complex network organization [92,101], positive adaptive changes in the function of the stress response network would, by necessity, foster an evolving cascade of additional adaptations and persistent positive changes across the rest of the organism, i.e., system-wide healing into greater resilience [21,22,145]. As we have previously proposed [21,22,237], the self-organized, interconnected network nature of the person [101] would underlie the clinically-reported pattern of homeopathic healing over time, i.e., from above downward, from more important to less important organs, and in reverse order in time of symptom appearance [238]. The pattern of clinical response usually begins in the brain because of its central role in interpreting and coordinating physiological responses of the body to perceived environmental threats and stressors. The global sense of well-being and symptom improvements at end organs would occur, but as an indirect and possibly temporally delayed, outcome of restoring allostatic network function to normal [136].

## Summary

In summary, the basic tenets of the model are [39]: Homeopathic remedies are remedy source nanoparticles and/or remedy-modified silica NPs that act as environmental stressors to mobilize hormesis and time-dependent sensitization via non-pharmacological effects on specific biological adaptive mechanisms. Both top-down mechanical attrition (trituration milling in lactose; succussion in glass with ethanol-water diluent) and plant-tincture biosynthesis methods generate the initial nanostructures. The nanoparticle nature of remedies distinguishes them from conventional bulk form drugs in structure, morphology, and functional properties. Furthermore, remedy source nanoparticles, especially in interaction with nanosilica, have the capacity to initiate bottom-up self-assembly of biomimetic nanostructures using crystalline or biological, e.g., DNA, proteins, collagen, templates [71,76,239]. Like a virus, albeit non-infectious, the homeopathic remedy thus becomes a salient low-level danger signal or threat to the survival of the organism.

The outcomes depend upon the ability of the organism to appraise the original high level stressors that caused disease and the subsequent low level remedy nanoparticles as novel and salient foreign stressors. Factors identified as biological threats will signal the need for time-dependent, sensitized compensatory adaptations (hormesis) in components of the allostatic stress response network.

The cumulative impact of allostatic overload from multiple different stressors led in the past to a pattern of specific dysfunctional adaptations in the stress response network underlying the emergence of disease [195,240]. The cross-adapted/cross-sensitized homeopathic remedy nanoparticles take advantage of the priming effect of the prior high level stressors that originally caused the disease [10]. The remedy nanoparticles, as a low level stressor, then elicit reversal of direction in the pre-established, disease-related maladaptive patterns. The net outcome is improved resilience to stress, with restoration of normal homeostatic function, resolution of disease, and an emergent sense of global well-being.

Again, “stress” refers to biological, infectious, chemical, physical, electromagnetic, nutritional and/or psychological types of environmental stimuli that the organism recognizes as a novel threat to its survival, now or in the future. The high or low intensity of the stressor determines the direction of the adaptations it initiates [98,187,216], but it is the encroachment of the stressor on the organism that mobilizes plastic and metaplastic changes. In short, perceived or experienced novel threat is more important than dose level to trigger adaptive responses. Dose comes into play to modulate the direction of the responses via priming from past cellular activity history, e.g., metaplasticity, and current plasticity in the body’s stress response pathways [181].

Within the organism as a complex adaptive system or network, causality for these events is indirect rather than direct, distant in time and space to the original administration of the homeopathic dose as a small but salient stimulus or stressor [241]. The organism carries forward the work of healing as a nonlinear, amplified dynamical adaptive response [21-23,145]. It is because of the nature of classical homeopathic prescribing, i.e., selection of a single remedy administered intermittently, at widely-spaced intervals of time in pulsed acute dosing regimens that the treatment system is safe and beneficial.

Table 1 summarizes the parallels between key homeopathic clinical concepts and concepts found in the basic science literature on nanoparticles, hormesis, time-dependent sensitization, allostatic adaptation, and complex adaptive systems.

**Table 1:** Parallels between homeopathic and modern scientific research literatures

**Homeopathic Literature**	**Relevant Modern Scientific Literature**
Disease is the manifestation of “dynamic mistunement” of the living system (life force) [40]	Disease is the current manifestation of failure to adapt or compensate for allostatic overload from convergence of biological, chemical, physical, and psychological stressors on the nonlinear adaptive stress response network, which is embedded within the larger complex network of the overall organism [95,144]
Homeopathic remedies are made with trituration and/or serial dilutions and succussions of source material, usually in glass containers, which generate nanoparticles of source and source adsorbed to silica nanoparticles in colloidal solution [1-3,24,64]	Nanoparticles can initiate hormetic low dose responses in the organism (adaptive or compensatory changes opposite in direction to the effects of the agent at higher doses) [16]
Remedies prepared and succussed in polypropylene or polyethylene vials could also have polymer-derived nanoparticles, but with different properties from those made in glass [1].	Nanoparticles have high surface to volume area and quantum-like properties. They differ from bulk source materials in exhibiting greater ability to translocate around the body and into cells, as well as increased catalytic activity, adsorptive capacity, and different electrical, magnetic, optical, and thermal properties from molecules of the “same” bulk material [33,48,53,60].
	Biological structures, e.g., DNA, proteins, or collagen, adsorbed to exogenous nanosilica and other specific nanoparticle structures, e.g., calcium phosphate or gold, serve as epitaxial templates for bottom up self assembly of new biomaterials [76,239,242]
Higher potencies (more dilution and succussion steps) have longer lasting effects on living systems [243] (succussion involves intense mechanical shaking of the solution by pounding the glass container against a hard elastic surface) Succussion in glass containers releases variable amounts of silica as nanoparticles [4]; remedy samples prepared in glass vs polypropylene containers differ in physico-chemical properties [1] Direction of effects of sequential remedy potencies can be nonlinear (oscillatory) in pattern [12]	Succussion, like modern microfluidization techniques [51], introduces cycles of fluid acceleration and turbulence with repeated changes in the direction of flow, producing the potential for particle collision and shear forces to break off smaller and smaller particles. These procedures, while different from each other and from sonication as a technique for agitating solutions and producing nanoparticles, share the ability to create nanobubbles and shear forces. Nanoparticle research suggests that there are nonlinear relationships between the number of microfluidization cycles or sonication time and variations in the sizes, morphologies, and physico-chemical properties of the “same” bulk-form material substance [52,53,244].
	Such data suggest the hypothesis that different amounts and forces of succussion should also generate different sizes, morphologies, and physico-chemical properties of homeopathic remedy source and remedy-modified silica nanoparticles [64].
	Silica [128,245] and polystyrene [246] nanoparticles are used in conventional nanomedicine as drug/gene delivery vehicles
	Direction of effects of sequential nanoparticle cluster sizes can be nonlinear (oscillatory) in pattern [48]
Pulsed dosing regimens of low doses (single or intermittent repetitions of remedy doses, widely spaced in time) exert persistent effects on physiology and behavior [13,14,154,159,243]	Low doses of nanoparticles can serve as highly reactive environmental stressors, not simply as pharmacological agents [187], for the organism to initiate allostatic adaptations over time. These endogenous changes compensate for and protect against other cross-adapted or cross-sensitized stressors (i.e., the adaptations already in place from the cumulative effects of disease-causing events *on the same components of the stress response network*) [5,95].
	Single or intermittent repetitions of low intensity levels of a foreign stressor or substance initiate a process of progressive endogenous response amplification over time (TDS, time-dependent sensitization) [98]
	At the physiological limits of the system, the direction of sensitized responses become nonlinear (oscillatory) and reverse direction in pattern [7,180,181,186]
In an intact person, patterning of remedy responses sometimes includes transient worsening (aggravations) and, when clinically successful, follows Hering’s Law of Cure (center of gravity of disease moves from top to bottom of organism; from more important to less important organs; and in reverse order of occurrence in time) [238]	Central nervous system pathways are a major hub for regulating the allostatic stress response network of the body, interacting with hubs of the immune, endocrine, and autonomic nervous system to generate the overall global and local patterns of responses across the organism to any type of environmental stressor [6,134].
However, homeopathic remedies can also exert measurable effects on living cells as complex adaptive systems or networks [7-9,12,108,147,173].	However, living cells are also complex adaptive networks unto themselves. As such, cell systems can self-reorganize their biochemical functional networks in response to a stressor such as heat shock without requiring the rest of a larger network or brain [93,142].
	Overcompensation of hormetic adaptations to a low level stressor can lead to salutary allostatic effects on the organism or complex adaptive network [247-249]
	Human beings are complex adaptive systems that are self-organized, with interactive global and local patterns of adaptive behavior that modify each other’s functional behaviors [21,23,94]

## Conclusions

The proposed model suggests that homeopathy is not only scientifically “plausible,” but also grounded in an extensive empirical research literature. Homeopathic remedies come into existence and exert their biological effects mainly as nanostructures. Physiology, not pharmacology, is the most relevant discipline for studying remedy nanoparticle actions (cf., [184,187]). This paper insists on logic and rationality, as well as open-minded thoughtfulness, in evaluating the scientific implications of a large body of interdisciplinary evidence that health researchers might not otherwise assemble to understand homeopathic remedies. As empirical data arise, it is quite likely that new evidence will lead to modifications of the present theory; such is the nature of scientific inquiry. Nevertheless, this model provides a rational starting place for a comprehensive research program on homeopathic remedy actions. The resultant findings on what homeopathic remedies are (highly reactive nanoparticles) and how they interact with complex living systems (as pulsed, low level doses of a salient and novel environmental stressor) could significantly advance the field as a valuable form of nanomedicine.

## Abbreviations

CAM: Complementary and alternative medicine; CAS: Complex adaptive system; FM: Fibromyalgia; NM: Nanometer; NP: Nanoparticle; TDS: Time-dependent sensitization.

## Competing interests

Dr. Bell is a consultant to Standard Homeopathic/Hylands Inc, a U.S.-based manufacturer of homeopathic medicines. This company did not provide any financial support for the paper or its publication costs, and none of the homeopathic studies cited here utilized their products.

## Authors' contributions

IRB performed the literature search on homeopathy and nanoparticles. IRB and MK jointly developed the complex adaptive systems aspects of the model. IRB wrote the first draft of the manuscript. MK revised the draft, identifying key concepts and organizing the manuscript around the assumptions and principles stated in the final draft. Both authors revised the manuscript in accord with reviewer feedback and approved the final manuscript.

## Pre-publication history

The pre-publication history for this paper can be accessed here:

http://www.biomedcentral.com/1472-6882/12/191/prepub
